# Unstable Non-isthmic Spondylolisthesis Following Unilateral Biportal Endoscopy Assisted Unilateral Laminotomy for Bilateral Decompression: A Case Report

**DOI:** 10.5704/MOJ.2111.025

**Published:** 2021-11

**Authors:** IS Son, SY Han, HJ Chung, JE Hong, MS Kang

**Affiliations:** 1 Department of Orthopedic Surgery, Jeju National University College of Medicine and Graduate School of Medicine, Jeju, South Korea; 2 Department of Neurosurgery, Yonsei Knee Spine Hospital, Seoul, South Korea; 3 Department of Orthopedic Surgery, Bumin Hospital Seoul, Seoul, South Korea

**Keywords:** non-isthmic spondylolysis, laminolysis, retroisthmic cleft, facet tropism, unilateral biportal endoscopy

## Abstract

Lumbar decompressive laminectomy is a standard treatment for degenerative lumbar spinal stenosis, but in some cases, can lead to iatrogenic spondylolysis and delayed segmental instability. Iatrogenic spondylolysis occurs in most cases in pars interarticularis, but rare cases have also been reported, pediculolysis in pedicle and laminolysis in lamina. Minimally invasive spine surgery (MIS) is known to have a low risk of developing these iatrogenic spondylolyses, and unilateral biportal endoscopy is the MIS that has been drawing attention. We present a case of a 72-year-old female who was diagnosed with L4-5 unstable non-isthmic spondylolisthesis and severe right central disc extrusion 10 weeks after UBE assisted unilateral laminotomy for bilateral decompression (ULBD) at the consecutive segments of L3-4 and L4-5. Pre-operative imaging studies revealed severe central stenosis without spondylolisthesis at L3-L4 and L4-L5 along with L4-L5 facet tropism. She was managed by anterior lumbar interbody fusion and cement augmented pedicle screw fixation, which resulted in the complete resolution of her clinical and neurologic symptoms.

## Introduction

Lumbar decompressive laminectomy is a standard treatment for degenerative lumbar spinal stenosis, but iatrogenic spondylolysis and delayed instability are known complications. Although the spondylolyses occur mostly in the pars interarticularis (PI), they can occur, although rarely, in the pedicle and the lamina, immediately dorsal to the inferior articular process (IAP); in the pedicular cleft as pediculolysis, and in the retroisthmic cleft as laminolysis^1^. Both isthmic and non-isthmic spondylolisthesis significantly impact surgical outcomes and increase the risks for revision surgery. In addition, even though it remains controversial, the risk of delayed instability increases in surgery involving more than three segments, axial facet angle more than 50°, and intervertebral disc height higher than 6.5mm^2^. However, minimally invasive surgeries (MIS) using the microscopic tubular technique, such as unilateral laminotomy for bilateral decompression (ULBD), are reported to reduce these complications and improve clinical outcomes. Unilateral biportal endoscopy (UBE) is one MIS technique with less damage on the paravertebral structures, reversible muscle injury, less postoperative pain, and faster recovery^3^.

The authors present a case with unstable non-isthmic spondylolytic spondylolisthesis with retroisthmic cleft following UBE-ULBD.

## Case Report

**Fig 1:**
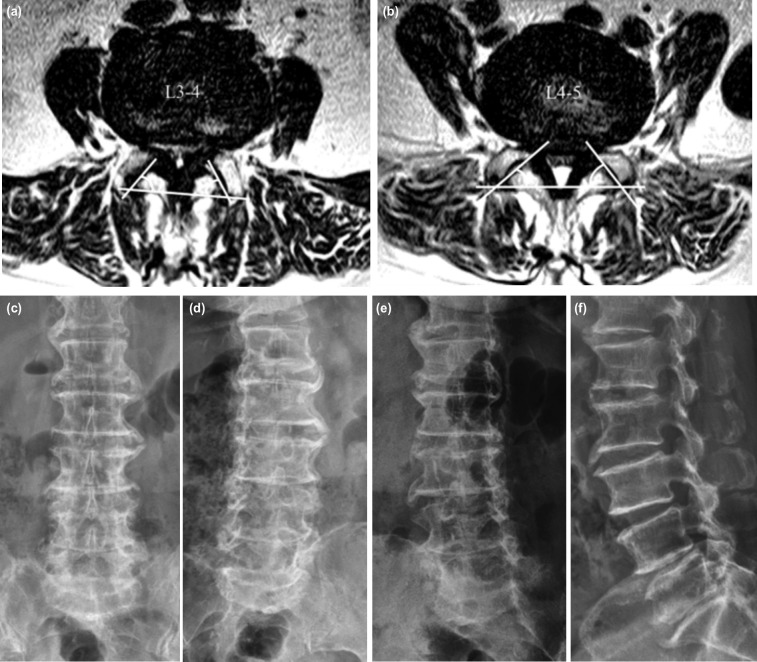
Pre-operative MRI and radiograph image. (a) L3-4 transverse and (b) L4-5 transverse MRI T2 weighted image shows moderate to severe central canal stenosis, and facet tropism at L4-5 (mean axial facet angle of 49°; 38° in right facet, 60° in left facet). (c) Standing AP. (d) Left oblique. (e) Right oblique. (f) Lateral radiograph shows disc height had been decreased to 6mm.

A 72-year-old woman visited our spine clinic, complaining of severe claudication, axial back pain and bilateral leg pain for more than one year of conservative treatment. Magnetic resonance imaging revealed moderate to severe central canal stenosis on L3-L4 and L4-L5 along with narrowed disc space (6mm) and facet tropism at L4-L5 (mean axial facet angle of 49°; 38° in right facet, 60° in left facet) (Fig. 1). Five months prior to the first visit to our clinic, she had undergone percutaneous epidural neuroplasty, but her pain symptoms returned within a month. Consequently, UBE-ULBD was performed at the consecutive segments of L3-4 and L4-5 two months after the primary operation. Postoperatively, she quickly recovered from her pain symptoms. However, within two weeks, she developed abrupt and progressively worsening radicular pain, muscle weakness, and gait difficulty on her dominant right leg. The pain worsened in standing and ambulation for less than a minute, which often required her to use a cane, in addition to intermittent bilateral leg cramping at rest without bowel and bladder problems.

**Fig 2:**
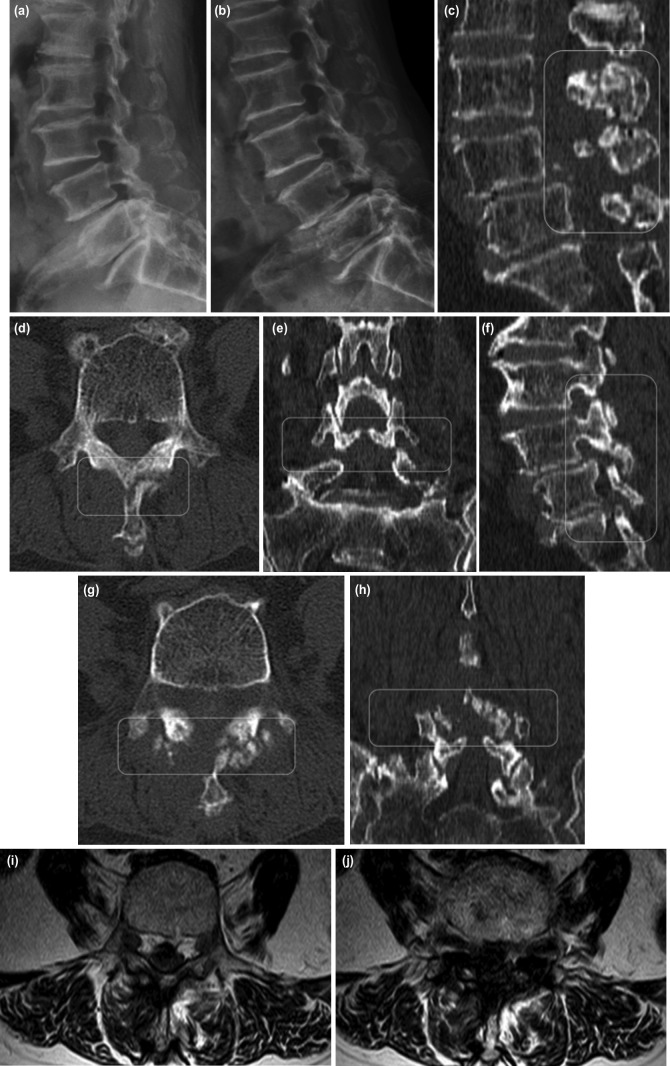
(a) Postoperative lateral radiograph at the time of BE-ULBD (b) and three months follow-up. (c, d, e) MRI and CT imaging for three months after the first surgery (BE-ULBD. Pre-operative CT cut showed a stress fracture of posterior arch at L3, (f, g, h) and L4 (white rounded rectangle). (i) Pre-operative T2 weighted MRI axial cut L4 spondylolytic spondylolisthesis, severe herniated nucleus pulposus and L3 and L4 posterior arch fracture. (j) Axial cut at L4-L5 level.

The straight leg raising test was unremarkable bilaterally. Neurologic examination revealed 4/5 tibialis anterior and dorsiflexion strength of the right ankle and 3/5 extensor hallucis longus and dorsiflexion strength of the right foot. Plantar responses were flexor without clonus. Lumbar standing radiographs before and after the primary UBE-ULBD showed no evidence of spondylolysis and spondylolisthesis (Fig. 1f and 2a). A follow-up lumbar lateral radiograph taken three months after the surgery, however, showed an L4 unstable spondylolytic spondylolisthesis (Fig. 2b). A follow-up MRI and CT showed a fracture of the spinolaminar junction from L3-4 to L4-5 and severe central stenosis due to recurrent lumbar disc herniation as well as contralateral laminolysis and spondylolisthesis at L4-5 (Fig. 2c-j).

**Fig 3:**
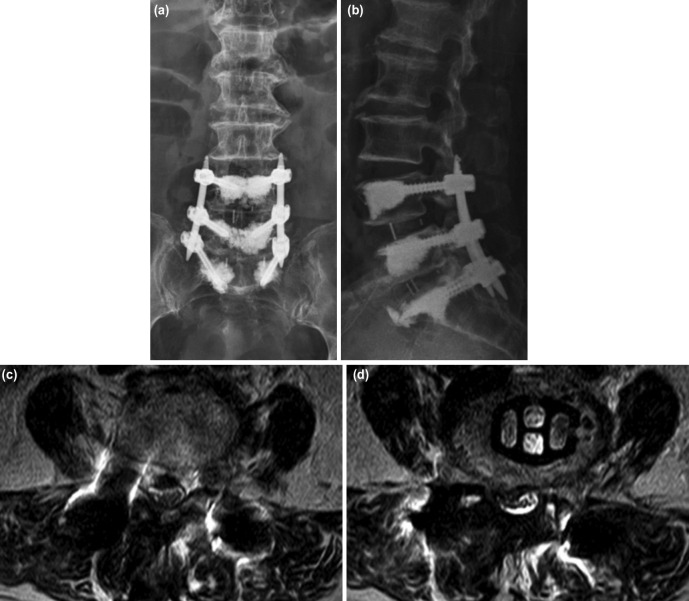
Postoperative radiograph and MRI image at the second surgery (ALIF). (a) AP, (b) lateral, (c) L4 posterior arch level, (d) an at L4-L5 level.

The patient underwent anterior lumbar interbody fusion (ALIF) and cement augmented pedicle screw fixation for the L4-L5 delayed segmental instability and the L5-S1 bilateral foraminal stenosis (Fig. 3). On the follow-up assessment at post-operative one year, the patient was back to full daily activities without axial back and leg pain.

## Discussion

This is a case report on delayed instability caused by non-isthmic spondylolisthesis with retroisthmic cleft that developed post-operatively 10 weeks after UBE-ULBD for severe central canal stenosis of the consecutive segments of L3-4 and L4-5 (Fig. 1). From a biomechanical point of view, delayed instability following lumbar decompressive laminectomy can be caused by a stress fracture of PI due to a decreased load bearing capacity of the intervertebral disc from compression-translation stress after discectomy and bilateral partial facet resection^4^. However, our case has several differences from the earlier studies. Without excessive facet joint resection or violation of the PI, spondylolisthesis occurred in the vertebral lamina immediately dorsal to the IAP, not in the PI. Without the presence of any risk factors for delayed instability, it was necessary to consider other factors that may have contributed to this condition.

In this case, despite failure in the spino-laminar junction in the two consecutive low lumbar segments, non-isthmic spondylolysis developed only in the lower segment. Delayed instability caused by contralateral pedicular cleft has been reported after decompressive laminectomy, and the cleft occurring in the pedicle and retroisthmic area is called ‘non-isthmic spondylolysis’^5^. These clefts are very rare and have been postulated to be congenital in origin or often the result of stress fractures. In particular, the orientation and shape of the facet joint are known to be related to the development of the spondylolisthesis because these affect the distribution of the load on the posterior column.

Asymmetric facet joints increase the force through one side of the functional segment, with unilateral spondylolysis occurring on the other side of the more coronally orientated facet joint and leading to unequal rotation in the axial plane, the condition of which is called ‘facet tropism’. These asymmetries are most often relatively subtle and amount to <5° in magnitude but may lead to the degeneration of the functional lumbar segment due to their contribution to weight-bearing on the lumbar spine1. Therefore, given the lack of radiologic risk factors and abrupt deterioration of neurological symptoms, it may be reasonable to assume that facet tropism in L4-5 caused a non-isthmic spondylolisthesis, increasing the load bearing capacity of the middle and posterior column for compression-translation load after ULBD. In this regard, the ALIF using a large lordotic interbody cage was able to recover the segmental kyphotic angle at the L4-L5 segment and to treat the moderate foraminal stenosis at L5-S1, along with the cement augmented pedicle screw due to the fragile bone quality.

Despite the clinical benefits of UBE-ULBD, the potential complications from altered spinal biomechanics should be considered. In particular, the facet tropism may be an independent factor causing delayed instability due to isthmic and non-isthmic spondylolysis. In addition, spine surgeons should pay attention to this potential complication and provide patients with clear information about it.
